# Temperature Influence on Brake Pad Friction Coefficient Modelisation

**DOI:** 10.3390/ma17010189

**Published:** 2023-12-29

**Authors:** Costanzo Bellini, Vittorio Di Cocco, Daniela Iacoviello, Francesco Iacoviello

**Affiliations:** 1 Department of Civil and Mechanical Engineering, University of Cassino and Southern Lazio, Via G. Di Biasio, 43, 03043 Cassino, Italy; v.dicocco@unicas.it (V.D.C.); iacoviello@unicas.it (F.I.); 2 Department of Computer, Control and Management Engineering, Sapienza University of Rome, Via Ariosto, 25, 00185 Roma, Italy; daniela.iacoviello@uniroma1.it

**Keywords:** brake pads, friction coefficient, tribological behaviour

## Abstract

Brake pad linings are an essential part of the correct functioning of braking systems based on the use of pads and discs. Generally, the compounds used to make the gaskets are characterised by the use of over 20 sintered components, which allow friction coefficients that vary between 0.2 and 0.6 at temperatures up to 200 °C. In this work, a traditional compound was investigated under close-to-real conditions in order to evaluate the tribological behaviour at different temperatures. Finally, a model based on the proportionality between temperature increase and relative variation of the friction coefficient was proposed. From the experimental test, it was evident that the friction coefficient increased with the temperature, passing from 0.4 to 0.6 in the temperature range of 100 °C to 180 °C; however, a further temperature increment until 350 °C caused a reduction in the friction coefficient to 0.2. The proposed model was able to anticipate the abovementioned trend, especially at high temperatures.

## 1. Introduction

The brake system is one of the most important pieces of equipment for the safety and correct functioning of cars and other means of transport. Among the various types of brakes, disc brakes currently represent one of the best technological solutions in terms of efficiency, ease of maintenance, and cost. The seals, which constitute the functional part of the friction system, are decisive regarding the performance of the entire braking system. In recent years, various compounds have been developed to create efficient brake pads at different temperatures of use, considering not only the friction coefficient but also its consistency under varying temperatures and braking conditions. In particular, various aspects have been optimised, ranging from noise under cold or humid conditions to thermal deformation, which could influence the strokes of the actuating clamps of the braking unit. Generally, brakes can be grouped into two main classes: brakes that work at low temperatures (typical in cars, for example) and brakes that operate at high temperatures (trains, trucks, buses, etc.). For decades, drum brakes with organic linings were the standard for passenger cars and light trucks. However, during the 1960s and 1970s, a shift towards disc front/drum rear brake configurations emerged. This marked the beginning of a rapid transformation period in brake technology. The 1970s were characterised by three significant challenges for the automotive industry: the need to phase out asbestos in friction materials, stricter directives for braking systems, and the transition to lighter front-wheel-drive vehicles for enhanced fuel efficiency. These factors involved the spread of a new class of friction materials [1].

The brake pad–disc system withstands continuous adhesive and abrasive wear, generating wear debris that poses environmental concerns. Conventional brake discs, typically made of grey cast iron, offer high thermal inertia and conductivity but are also prone to corrosion and significant wear. In fact, about 50% of total non-exhaust wear emissions from vehicles are provoked by PM10 emissions from brake wear. The interaction between friction pairs is crucial in determining braking performance under various conditions. The friction material used in brakes significantly affects the contact behaviour in the brake disc, making its properties critical for brake capabilities, especially under critical braking circumstances. The sensitivity of disc brakes to temperature, pressure, and speed during the braking phase is primarily influenced by the system constituents and their proportions. The historical development of brake pads, the challenges faced by the brake pad–disc systems, and their potentialities were studied in [2]. The composition of the pads has also been studied. For example, phenolic resins are commonly employed as binders in friction composites, but they have several drawbacks, such as the emission of dangerous gases during manufacturing, short shelf life, and the necessity to add a curing agent before shipping. To address these issues, some resins have been developed and evaluated as a potential replacement for traditional phenolic resins [3]. For example, a monomer polymerised by temperature-activated ring opening was developed and utilised as a binder in a friction composite. In particular, two kinds of non-asbestos organic (NAO) friction composites were considered, and their thermal, physical, and mechanical properties were characterised by testing their fade and recovery characteristics according to the ECR 90 standard.

Despite extensive research on the causes of automotive brake squeal, the exact mechanisms remain elusive. Some studies have delved into the underexplored connection between the brake pad finish and the squeal phenomenon. For example, Eriksson et al. explored the behaviour of two sets of brake pads, both made of a metal–fibre organic composite, which were subjected to brake tests in a dedicated test rig [4]. One pad was made of a standard production formulation, while the other was slightly modified for enhanced high-speed performance. The surface topography of the brake pads was analysed after stopping the tests in both silent and squealing states. By comparing these pads, the authors investigated the correlations between squeal, the number and size of contact plateaus, and the total area of contact. The findings suggested that a higher number of smaller contact plateaus and a reduced total contact area are associated with a higher propensity for squealing. Some other researchers used advanced microscopy and surface analysis techniques to study the chemical and microstructural variations on the surface of a conventional brake pad during braking simulations [5]. They found that parts of a third body material formed, consisting of a mixture of all the pad’s components and iron oxides from the brake disc. Debris particles from the pad retained the crystal structure of barite (the main phase of the pad material), but their grain size was dramatically decreased to the nanometric scale. The main wear process was the detachment of filler fragments from the organic binder, aided by localised degradation of the phenolic resin caused by heating. Quartz crystals remained intact and acted as primary contact areas.

For high-speed applications, the composition of the brake linings includes some metallic components. Copper-based composites manufactured using powder metallurgy processes have been demonstrated to be among the best materials for this purpose, and they are essential for high-speed trains travelling over 300 km/h [6]. However, some factors considerably affect the braking capacity, such as the manufacturing process, chemical composition, microstructure, and operating conditions. The underlying mechanisms of wear loss and thermal fade under thermal and mechanical coupling have not yet been fully elucidated. Recently, machine learning algorithms have attracted general attention and achieved remarkable results in materials science. Algorithms have been introduced to determine the braking capabilities of copper-based brake pads. A new copper-based alloy was defined by modelling the coefficient of friction (COF) and the wear loss. Braking tests demonstrated that the average COF of this alloy was 0.37 in the stable stage (I), and the thermal recession diminished by only 0.012 in stage II under continuous braking at 350 km/h and 0.48 MPa for 10 cycles. The total wear loss was only 1.30 g when braking from 200 km/h to 350 km/h with pressure from 0.31 MPa to 0.48 MPa. In contrast to commercial brake pads, the tested pads manifested excellent braking capabilities, with a 21% reduction in the thermal-fade COF and a 20% reduction in total wear loss. These results suggest that the optimised brake pad material could significantly improve the braking performance of high-speed trains.

It is difficult to control the wear and friction properties of potential brake pad materials for high-speed rails; thus, simulative testing techniques are needed. A braking testing procedure was developed to evaluate the effects of three different forms of iron-containing powders in copper-based composites on a computer-controlled dynamometer [7]. The brake pad with 30.6 wt% copper-coated iron powder presented the most elevated and stable friction coefficient of all tested composites, even after some emergency braking trials with a total absorptive energy of up to 32 MJ. At the end of simulated high-speed train braking, the friction coefficient of these pads was within the limits. Moreover, the brake pad wear loss was 0.25 cm^3^/MJ, which is 29% lower than the specified limit of 0.35 cm^3^/MJ. To use less copper, the industrial sintering method for making metallic matrix pads was modified [8]. Regretfully, it appears that there is a limit to how much iron can be used to replace copper. The materials were employed to the absolute limit in the high-energy, emergency-type rail brakes used in an investigation conducted by Mege-Revil et al., allowing for the observation of the evolution of the mechanical properties and microstructure of sintered metallic matrix pads. Following the braking test, digital image correlation (DIC) was used to evaluate their compressive behaviour, and scanning electron microscopy (SEM) was used to evaluate their microstructure. Three flat layers with various microstructures and compressive characteristics constituted the worn material. The bottom layer appeared unchanged, while the intermediate layer had microscopic and macroscopic fissures ranging in depth from 2 to 15 mm. Due to the resolidification of copper, the top layer became more rigid. During the braking test, the temperature rose to 1000 °C, which also clarifies how the iron-graphite contacts in the pad were weaker due to carbon diffusion into iron. Lastly, several open interfaces of the crushed and eroded pad showed the presence of submicronic particulate matter.

Chen et al. examined the wear processes and braking tendencies of Cu-based PM brake pads mated with 30CrMnSi steel discs and a C/C-SiC steel discs during high-energy braking scenarios [9]. According to the results, the wear rate and braking distance of the PM C/C-SiC pad were lowered by 31.0% and 29.5%, respectively, while the COF and the stability coefficient were raised by 28.9% and 13.9%, respectively, in comparison to the PM-30CrMnSi brake pair. Additionally, the PM-C/C–SiC brake pair did not exhibit significant tail warping in the braking curve, which may have been caused by the lubrication from carbon fibres and the generation of a dense tribolayer. The main wear process for the PM-C/C–SiC brake pair was oxidation and delamination wear, while for the PM-30CrMnSi brake pair, the main process was adhesive and abrasive wear. While there are not many options to change the nature of the material used to make discs, there is a nearly limitless amount of options when it comes to pads. To achieve the desired outcome, manufacturers and scientists have created and experimented with different combinations. Borawski investigate whether copper could replace the materials often utilised as reinforcing materials, given that these materials are often associated unfavourable risks throughout the production process [10]. To find the abrasive wear rate and the COF, tribological experiments were performed on a number of samples that included copper in the form of both powder and fibres. The testing procedure was designed using the Taguchi optimisation approach and the ball-cratering research method. The test findings indicated that the abrasive wear rate and COF value were not considerably impacted when copper fibres were substituted for aramid fibres.

Other works investigated how the grain size of the copper matrix in sintered copper-based composites affected the microstructure and tribological properties of the tribolayers formed during sliding friction [11]. The results showed that the wear dynamics and the composite hardness had an important impact on the stability and composition of the tribolayers. Electron microscopy revealed that the tribolayers are composed of either iron oxides or copper, with varying areal coverages of an amorphous phase surrounding them. The tribolayer self-lubricating characteristics were found to play a crucial role in determining the tribological peculiarities, such as the wear resistance and the friction coefficient. Therefore, the optimal sintering setup for copper-based composite manufacturing was determined to be 800 °C and 40 MPa. Composites sintered according to these parameters presented an ultrathin and robust tribolayer with a high carbon content.

Because of the complexity and variability of their operating settings, little is known about the friction performance of sintered brake pads made of copper, for which wear rate prediction is challenging. Thus, using an atom search optimization backpropagation neural network to estimate the wear rate of a Cu-based PM brake pad, the variation trend of the brake pad’s friction performance under various multifactor coupling braking situations was investigated by Chen et al. [12]. Cu-based brake pads are considered a research object whose wear rate can be predicted using an atom search optimization backpropagation neural network obtained by optimizing the weights and thresholds of the back propagation neural network using atom search optimization method. The wear rate and other brake features were examined under multifactor coupling braking conditions. The findings indicated that the primary determinants of braking performance are braking speed and braking pressure. The mean friction coefficient of the Cu-based brake pads ranged between 0.35 and 0.45 under various braking circumstances.

Under severe braking circumstances, the temperature-dependent evolving friction and wear characteristics of a copper-based pad on a steel brake disc were examined by Le et al. [13]. Their findings show that while there was some unpredictability in the friction and wear behaviours of the pad as the temperature rose, there was a clear general tendency: at room temperature, the friction coefficient was 0.33; then, it increased as the temperature rose over 170 °C and reached its maximum value at 240 °C. The friction coefficient was constant until 400 °C; then, it declined drastically. As regards the wear rate, it began to grow substantially as the temperature approached approximately 100 °C, and at 500 °C, it increased by an order of magnitude in comparison to room temperature.

Singh created automotive brake pads using various ratios of cement bypass dust and barium sulphate, and their tribological characteristics were assessed using a Krauss machine in accordance with European requirements [14]. When the abovementioned substances were added in increments of 0 to 30 weight percent, the friction coefficient rose; however, it decreased with a further content increment. On the other hand, as cement by-pass dust concentrations rose and barium sulphate levels decreased, wear and friction fluctuations increased. The composite containing 50 wt% cement bypass dust demonstrated the highest fade resistance of 15.36%. A study of the effects of wear and friction showed that while material stability and fade reaction were important factors in determining wear performance, fade and recovery response determined the entire friction performance.

Carbon–carbon brake disks are particularly sought after for use in trucks, race cars, trains, and airplanes. However, their vulnerability to oxidation in the presence of oxygen at temperatures around 400 °C is a fundamental limitation, in contrast to their advantageous mechanical and thermal properties. An experimental investigation conducted by Sunil Kumar et al. showed how oxidation in static air affects carbon–carbon fracture toughness [15]. A temperature evaluation of the material oxidation was performed from 400 °C to 700 °C with increments of 100 °C. According to the results, the temperature increase from 400 °C to 700 °C resulted in a considerable drop in fracture toughness. It was noted that oxidation significantly influenced the fracture toughness of carbon–carbon material.

In the present work, mixed lining pads characterised by the presence of components that work better at low temperatures and components that operate better at high temperatures were investigated. The pads were realised using the same sintering conditions, and the tests were performed using a friction machine that was able to measure both the friction coefficient and the temperature during braking at a constant speed (40 km/h). A simple model was then proposed to predict the friction behaviour of pads at different temperatures within the working range. Various studies of the behaviour of braking pads made of different materials have been reported in the literature, but there is a lack of models suitable for predicting the friction coefficient trend relative to temperature.

## 2. Experimental Methods and Results

In order to evaluate the friction coefficient of the pad mix, avoiding the influence of the pad geometry, a circular shape of the pads was chosen. The pads are characterised by a flat-ring braking surface with a reduced width, allowing for the presence of debris during the braking operations. The pads were made of different materials, such as rubber and graphite, the performances are better at low temperatures, and glass and brass fibres, which operate better at high temperatures.

**Figure 1:**
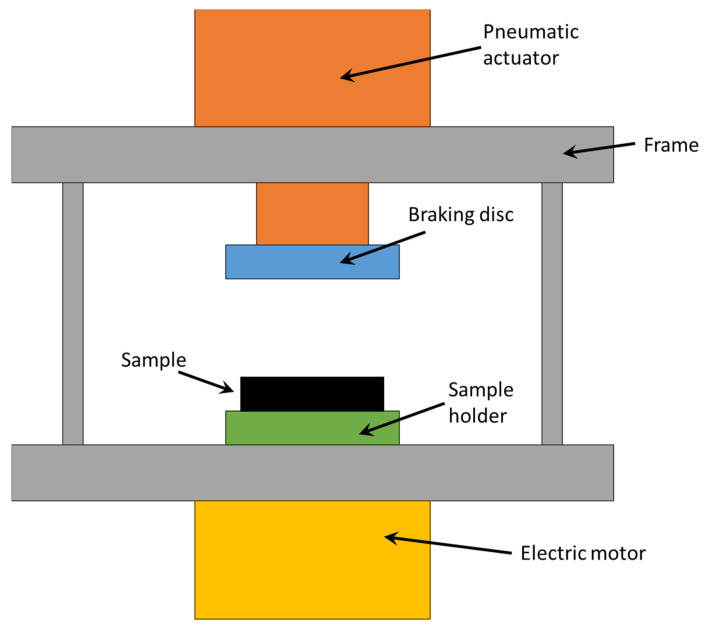
Equipment for braking tests.

The test equipment consisted of a frame on which a pneumatic cylinder, a braking disc, and a specimen holder were fitted. The pneumatic cylinder had a diameter of 160 mm and a stroke of 100 mm, and it was actuated and controlled by an electronic pneumatic system in order to ensure the desired contact force between the sample and the braking disc. This latter was connected to the pneumatic cylinder, while the specimen holder was connected to an electric motor and rotated at 420 RPM. The holder was made of Bakelite to reduce the effects of heat dissipation through contact with the brake actuating systems. A schematisation of the test equipment is shown in Figure 1.

**Figure 2:**
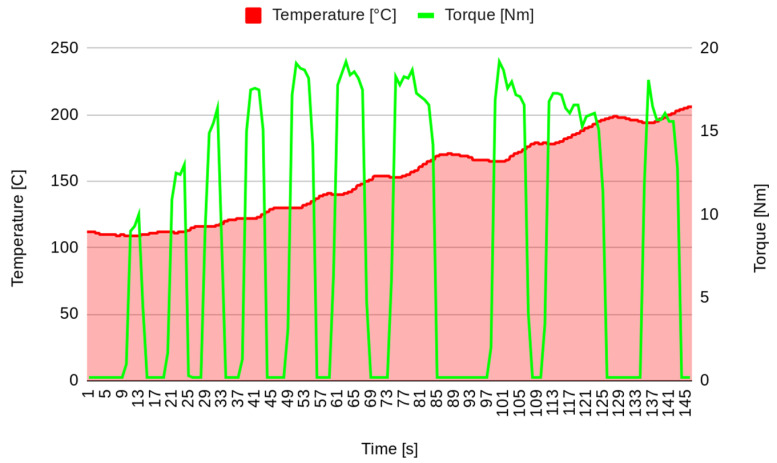
Pad surface stabilization (stage 0).

Prior to measuring the friction behaviour, some braking stages were performed to obtain a friction surface under normal braking conditions (Figure 2). The experiments were started on some brakes in order to stabilise the friction surface, achieving a temperature of 200 °C.

**Figure 3:**
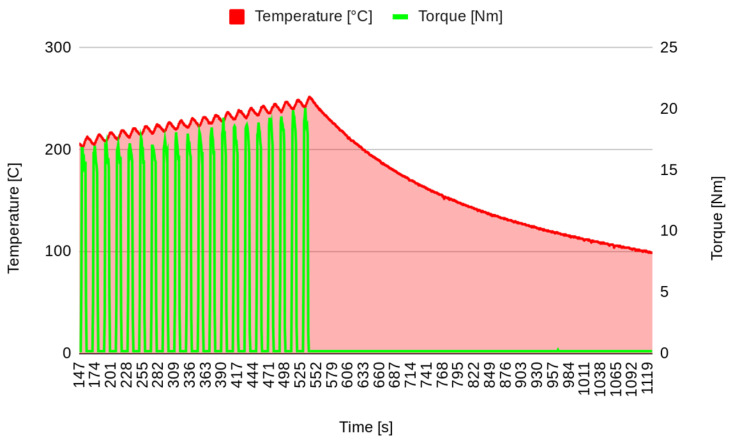
Stepping brakes (stage 1).

**Figure 4:**
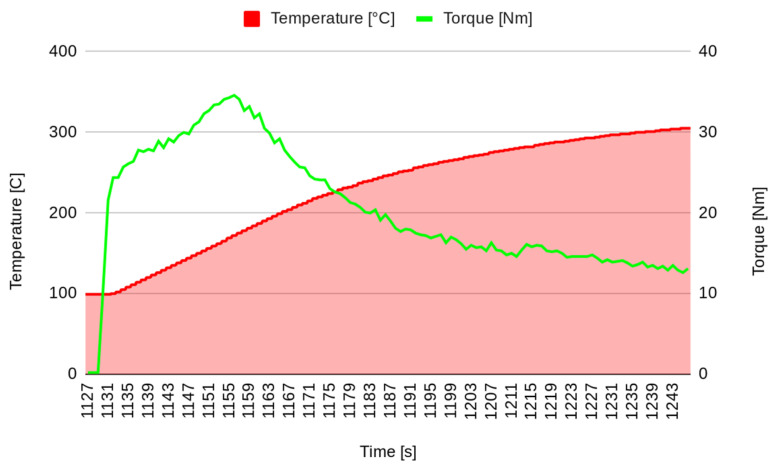
Continuous braking to achieve 300 °C (stage 2).

**Figure 5:**
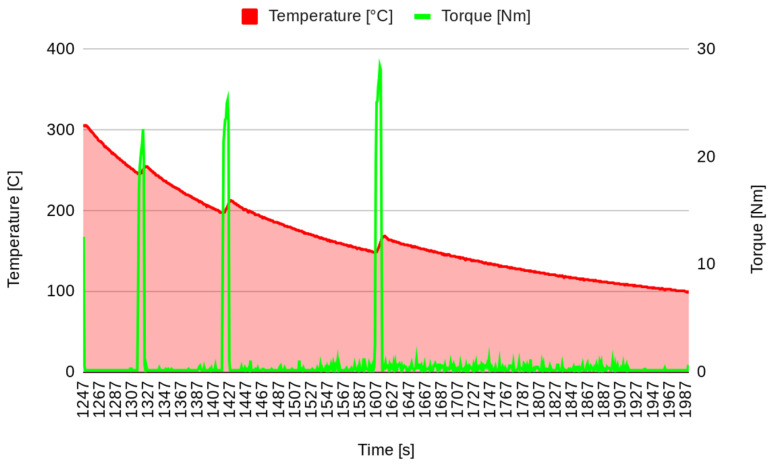
Cooling and cleaning (stage 3).

**Figure 6:**
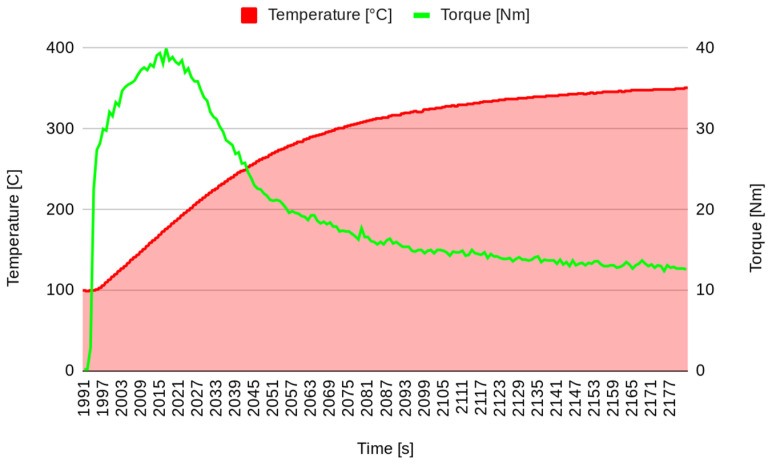
Investigated continuous brake (stage 4).

**Figure 7:**
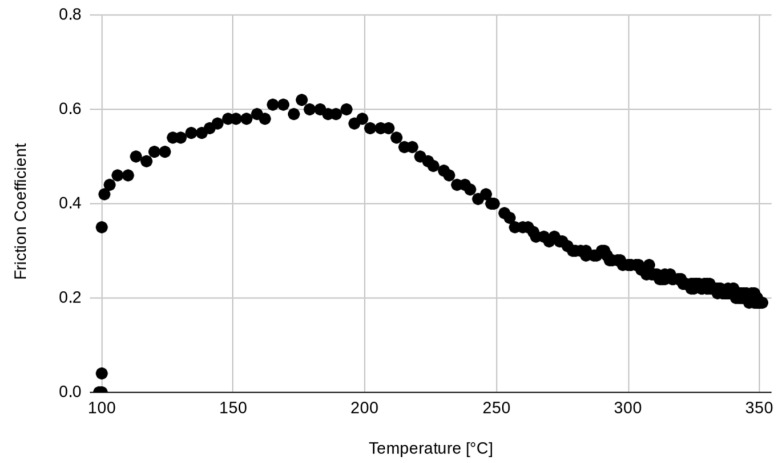
Influence of the temperature on the friction coefficient.

In the first stage of the experimental test, after surface stabilization, 20 brakes of 10 s were performed, separated by 10 s, simulating the intermittent braking typical in city driving. The temperature increased to over 250 °C (Figure 3), and then the system was left to cool down. The second stage started when the temperature decreased to 100 °C. In the second stage, continuous braking was performed in order to achieve a temperature of 300 °C (Figure 4). Then, the brake was stopped, allowing the pad to cool down to 100 °C (Figure 5). It must be underlined that the time reported on the horizontal axis of all the graphs is counted from the start of the test, so it does not start from zero but from the last instant of the previous phase. Continuous braking is sometimes typical when travelling downhill on roads, for example, from mountains. During cooling, three brakings of 10 s were performed at 250, 200, and 150 °C to eliminate the debris produced at this stage (Figure 5). In the fourth stage, another continuous braking was performed, allowing the temperature to increase up to 350 °C (Figure 6). In this last stage, the friction performance of the pads was analysed by plotting the friction coefficient on the achieved temperature, as shown in Figure 7. It can be noted that the friction coefficient trend as a function of the temperature presented an increasing trend from 100 °C until reaching the maximum value of 0.62 at 175 °C. Then, a decrease in the friction coefficient can be highlighted, with a higher rate until 250 °C, while in the last temperature range, the decrement speed is lower. A similar trend has been reported in other works, such as those by Wei et al. [16] and Iyida et al. [17].

**Figure 8:**
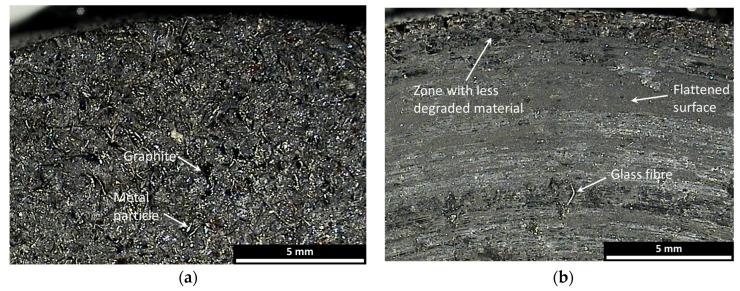
Micrographs of the pad surface: (**a**) before test; (**b**) after test.

The different morphology of the pad constituents before testing can be seen on the pad surface. In Figure 8a, the darker zones represent the components that work at low temperatures, while the clearer ones represent those that work best at high temperatures. A comparison of the pad surface before and after the test (Figure 8) evidences the damage to elements working at low temperatures, which are flattened on the centre of the pad. Along the external zones, the surfaces seem to be similar to the untested surfaces. This suggests a greater expulsion capacity of degraded material due to the elimination of debris during braking. In the central zone, it is possible to note the presence of a glass fibre.

## 3. Model Formulation

The components present in the investigated pads were chosen to allow for good tribological behaviour at different temperatures. There are some components, such as rubber or graphite, that work well at low temperatures, and other that are used at high temperatures, such as brass particles or glass silicates.

Considering the class of components working at low temperatures, a simple model to correlate the variation in temperature with the variation in the friction coefficient can be expressed as (1):

(1) $$\beta \cdot dT=\frac{{d\upsilon }_{L}}{\upsilon }$$

where *β* is a coefficient that depends on the mix components and their sintering conditions, *dT* is the variation in temperature during the break, and dυ_L_/υ is the variation in the friction coefficient with respect to the friction coefficient value.

By integrating (1), (2) can be obtained as:

(2) $$\int \beta \cdot dT=\int \frac{{d\upsilon }_{L}}{\upsilon }\to \beta \cdot dT+a=ln\upsilon_{L}$$

This means that the friction coefficient can be expressed as (3) by inverting (2) as follows:

(3) $$\upsilon_{L}\left(T\right)=\alpha \cdot e^{\beta T}$$

Similarly to low-temperature working components, for high-temperature components, the contribution to the friction coefficient is expressed as (4):

(4) $$\upsilon_{H}\left(T\right)=\gamma \cdot e^{\delta T}$$

Finally, the friction coefficient is the result of the summation of the single contribution coefficients (5):

(5) $$\upsilon \left(T\right)=\upsilon_{L}\left(T\right)+\upsilon_{H}\left(T\right),$$

and the final relation between the friction coefficient and temperature is expressed as (6):

(6) $$\upsilon \left(T\right)=\alpha \cdot e^{\beta T}+\gamma \cdot e^{\delta T}$$

## 4. Validation of the Model

**Figure 9:**
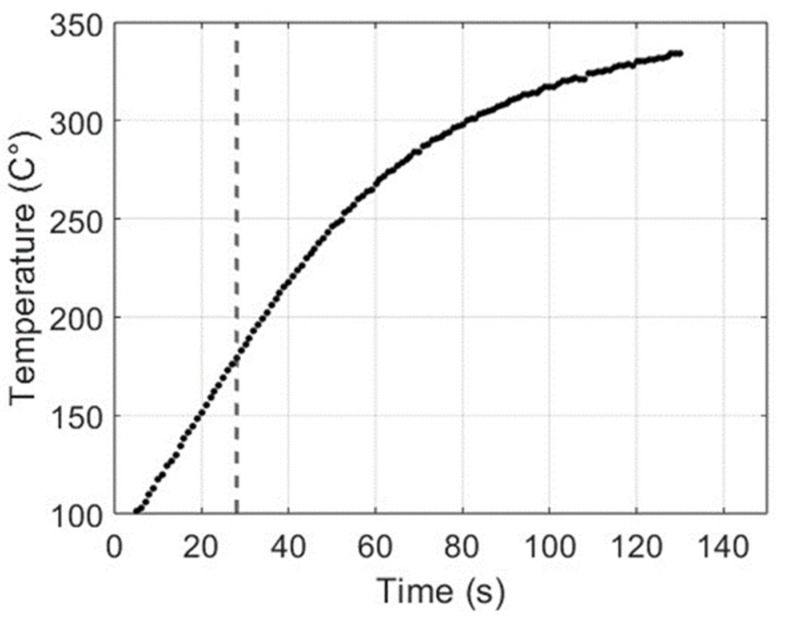
Representation of the temperature variation after the preparation phase.

**Figure 10:**
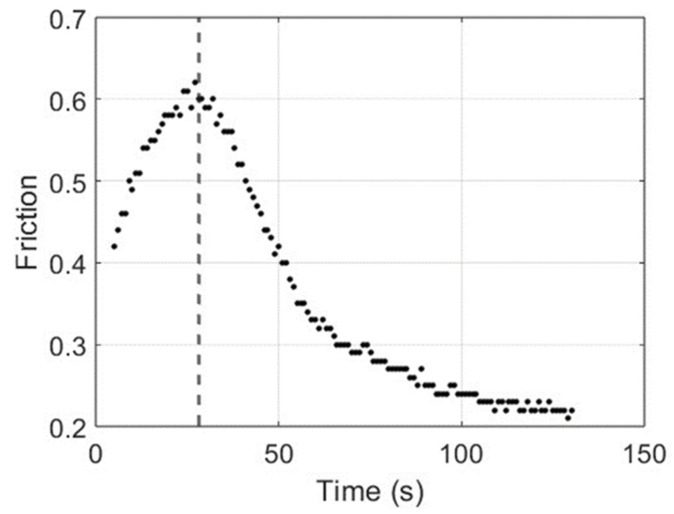
Representation of the friction evolution after the preparation phase.

The data considered in this analysis are the temperature (T) and the friction (F) collected at the same instants after a preparation phase in which the brakes were stressed with braking and cooling actions. Figure 9 shows a representation of the increasing temperature, whereas the variation in friction is presented in Figure 10.

**Figure 11:**
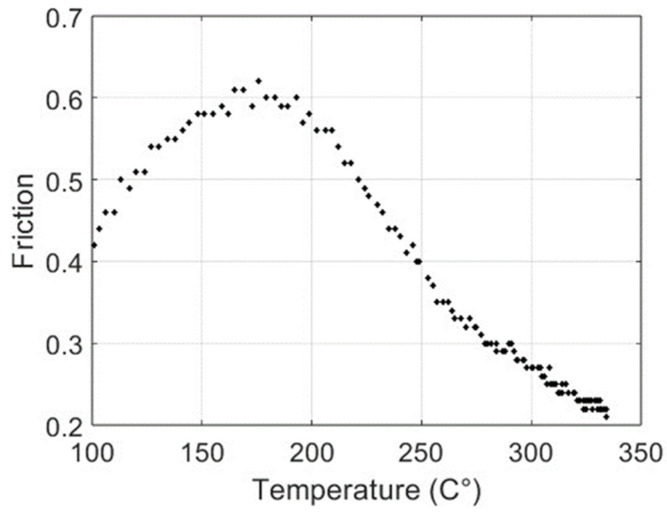
Representation of the couples (T, F).

Note that up to a temperature of T = 193 °C, the friction increases and shows monotonically decreasing behaviour. It is interesting to study the relation between temperature and friction. In Figure 11, the couples (T, F) are shown; each point represents the values of the temperature (in the abscissa) and the friction (in the ordinate) at the same instant.

The non-monotonic behaviour of the friction as temperature increases probably depends on the behaviour of the components in the pad mix.

Considering (6), the problem of determining the coefficients ((***α***, ***β***, ***γ***, ***δ***) ∊ ℝ^4^) can be solved by minimising the quadratic error between the proposed model and the collected data (*F*) as:

(7) $$J\left(\alpha ,\;\beta ,\;\gamma ,\;\delta \right)={min}_{\left(\alpha ,\;\beta ,\;\gamma ,\;\delta \right)\in R^{4}}\sum_{i=1}^{N}{\left(y\left(i\right)-F\left(i\right)\right)}^{2}$$

where *N* is the amount of available data.

Numerically, minimisation is solved by means of the Levenberg–Marquardt algorithm [18], an iterative method that implements the strengths of the gradient descent algorithm (slow but converging) and the speed of the Gauss–Newton solver.

**Figure 12:**
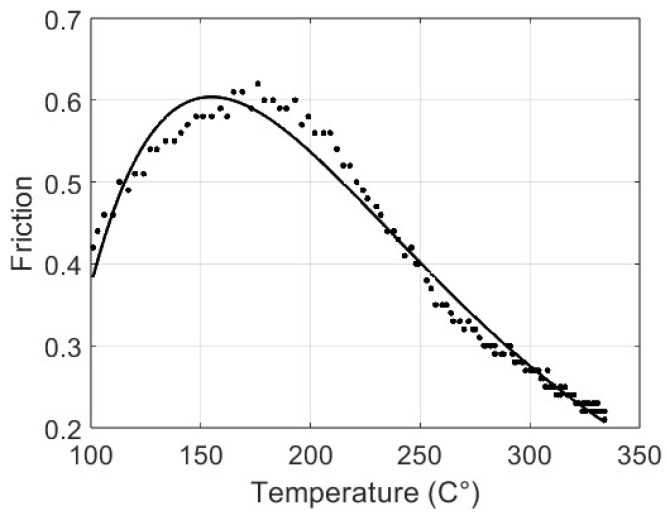
Original data (F(T)) versus the identified fitting curve (f(α, β, γ, δ)).

In Figure 12, the continuous line represents the function (f) with the identified parameters: *α* = 258,672.887873, *β* = −0.0125563826, *γ* = −258,676.877608, and *δ* = −0.012556587.

**Figure 13:**
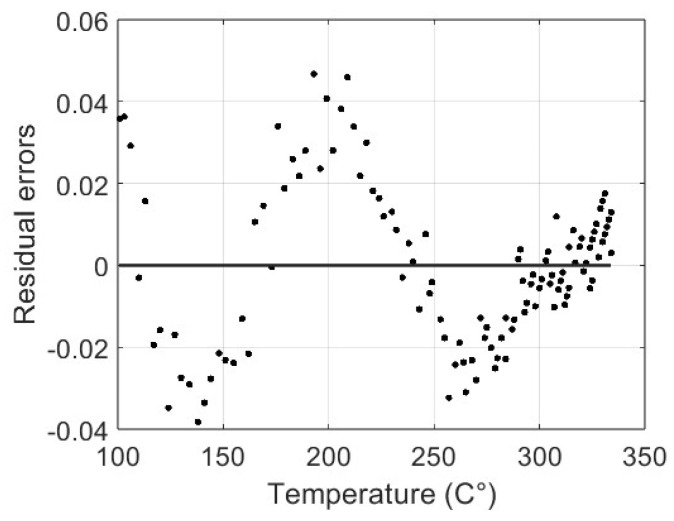
Residual errors between the original data and the identified fitting curve.

The sum-of-squares error is 0.041, with a root mean square error of 0.018. In Figure 13, the graph of the residual errors is shown; note the higher values at about 200 °C. These results attest to the capacity of the proposed model to anticipate the friction coefficient based on temperature data, even if a slight inaccuracy can be noted at lower temperatures.

## 5. Conclusions

In this work, a traditional compound, containing components that work at low temperatures, such as rubber or graphite, and high temperatures (brass and glass fibres) was investigated under close-to-real braking conditions.

These conditions were simulated by carrying out a series of braking cycles to increase the temperature of the new compound up to 200 °C, followed by a series of intermittent and continuous braking cycles before carrying out continuous braking until reaching 300 °C. During this last phase, the braking torque and temperature were monitored in real time in order to calculate the friction coefficient and plot its dependence on temperature on a graph.

The behaviour indicated an increase in the friction coefficient from 0.4 to 0.6 in the 100–180 °C range and a decrease to 0.2 at high temperatures (300 °C).

Finally, a model capable of evaluating the tribological behaviour of a brake pad compound at different temperatures was proposed. The proposed approach relates the temperature variation of the pad compound to the relative variation in the friction coefficient according to a combination of exponential laws.

## Data Availability

The data presented in this study are available upon request from the corresponding author.

## References

[1] Jacko M.G., Tsang P.H.S., Rhee S.K. (1984). Automotive friction materials evolution during the past decade. Wear.

[2] Mulani S.M., Kumar A., Naiyer H., Shaikh E.A., Saurabh A., Singh P.K., Verma P.C. (2022). A review on recent development and challenges in automotive brake pad-disc system. Mater. Today Proc..

[3] Gurunath P.V., Bijwe J. (2007). Friction and wear studies on brake-pad materials based on newly developed resin. Wear.

[4] Eriksson M., Bergman F., Jacobson S. (1999). Surface characterisation of brake pads after running under silent and squealing conditions. Wear.

[5] Österle W., Griepentrog M., Gross T., Urban I. (2001). Chemical and microstructural changes induced by friction and wear of brakes. Wear.

[6] Wu L., Zhang P., Xu B., Liu J., Yin H., Zhang L., Jiang X., Zhang C., Zhang R., Wang Y. (2023). Data-driven design of brake pad composites for high-speed trains. J. Mater. Res. Technol..

[7] Zhang P., Zhang L., Fu K., Cao J., Shijia C., Qu X. (2018). Effects of different forms of Fe powder additives on the simulated braking performance of Cu-based friction materials for high-speed railway trains. Wear.

[8] Mege-Revil A., Rapontchombo-Omanda J., Serrano-Munoz I., Cristol A.L., Magnier V., Dufrenoy P. (2023). Sintered brake pads failure in high-energy dissipation braking tests: A post-mortem mechanical and microstructural analysis. Materials.

[9] Chen F., Li Z., Luo Y., Li D.J., Ma W.J., Zhang C., Tang H.X., Li F., Xiao P. (2021). Braking behaviors of Cu-based PM brake pads mating with C/C–SiC and 30CrMnSi steel discs under high-energy braking. Wear.

[10] Borawski A. (2023). Study of the influence of the copper component’s shape on the properties of the friction material used in brakes—Part one, tribological properties. Materials.

[11] Lee H., Kim K.I., Kim J., Pin M.W., Oh K.H., Kim K.T. (2022). Electron microscopy characterization of the tribolayer formation mechanism in sintered Cu-based composites under dry sliding. Mater. Today Commun..

[12] Chen J., Yu C., Cheng Q., Guan Y., Zhang Q., Li W., Ouyang F., Wang Z. (2023). Research on friction performance and wear rate prediction of high-speed train brake pads. Wear.

[13] Lu C., Jiang X., Chen X., Mo J. (2022). Experimental study on the evolution of friction and wear behaviours of railway friction block during temperature rise under extreme braking conditions. Eng. Fail. Anal..

[14] Singh T. (2023). Comparative performance of barium sulphate and cement by-pass dust on tribological properties of automotive brake friction composites. Alex. Eng. J..

[15] Sunil Kumar B.V., Londe V.N., Lokesha M., Vasantha Kumar S.N., Surendranathan A.O. (2021). Influence of oxidation on fracture toughness of carbon-carbon composites for high-temperature applications. Fract. Struct. Integr..

[16] Wei Y., Wu Y., Chen K., Sun A. (2018). Experiment research on friction coefficient between a steel plate and rail in transient sliding thermal contact through a pendulum. Results Phys..

[17] Iyida B.U., Nwankwo A.M., Onah T.O. (2023). Parametric Effects on the Coefficient of Friction of a Novel Composite Material for Automobile Brake Linning. Int. Res. J. Multidiscip. Technovation.

[18] Kumaraswamy B., Binu D., Rajakumar B.R. (2021). Neural networks for data classification. Artificial Intelligence in Data Mining: Theories and Applications.

